# The Use of Spirulina Powder to Enrich Honey with Antioxidants and Highly Digestible Protein

**DOI:** 10.3390/ijms27041941

**Published:** 2026-02-18

**Authors:** Małgorzata Dżugan, Karolina Trybulec, Ewelina Sidor, Michał Miłek, Monika Tomczyk

**Affiliations:** 1 Department of Chemistry and Food Toxicology, Faculty of Technology and Life Sciences, University of Rzeszów, Ćwiklińskiej 1a, 35-601 Rzeszow, Poland; 2 Ivmax Sp. z o.o., Sniadeckich 20d/7, 35-006 Rzeszów, Poland

**Keywords:** rapeseed honey, enrichment, *Limnospira platensis*, antioxidant properties, in vitro digestion, storage stability, bioaccessibility

## Abstract

Despite the presence of numerous bioactive compounds, honey is naturally low in protein, which limits its nutritional value. Adding spirulina powder, abundant in protein and antioxidants, seems to be a good way to increase the nutritional value of honey. Two commercially available dietary supplements of spirulina were used to enrich rapeseed honey (0.15–1.2%) during the creaming process. Obtained products were analyzed after 1, 3, and 6 months of storage at room temperature regarding basic physicochemical parameters, protein and phenolic content, and antioxidant capacity. Moreover, the bioaccessibility of antioxidants, as well as the protein digestibility, was assessed using an in vitro digestion model followed by sodium dodecyl sulfate polyacrylamide gel electrophoresis (SDS-PAGE). The study demonstrated that 1.2% enrichment of blue spirulina significantly increased the easily digestible protein content and antioxidant capacity. All of the tested parameters were maintained during storage. Spirulina-enriched honey can be recommended as a novel functional product with unique sensory features and evidenced health-promoting properties and can be a valuable dietary supplement.

## 1. Introduction

Global health challenges, such as pandemics, the rising incidence of lifestyle-related diseases, and increasing public awareness of healthy eating, have become major factors driving the functional food market. Although there is no universally accepted definition, according to regulatory and scientific bodies such as the European Food Safety Authority (EFSA), the U.S. Food and Drug Administration (FDA), and the World Health Organization (WHO), functional foods may be either naturally occurring or intentionally modified to enhance their health-promoting properties [1,2]. Such benefits are typically associated with the presence of bioactive compounds that support immune function, reduce oxidative stress, and prevent chronic diseases. Consequently, consumer demand for innovative, health-oriented products is steadily increasing. The Business Research Company reports that the global functional food market was estimated at USD 329.65 billion in 2023 and is projected to reach USD 586.06 billion by 2030 [3]. 

Varietal honeys are characterized by a relatively stable chemical composition dominated by carbohydrates and water. Simple sugars, primarily fructose and glucose, account for approximately 80–85% of honey’s total weight, while water content typically ranges from 16 to 20%, depending on floral origin, climate, and storage conditions [4,5,6]. From a nutritional standpoint, honey can be described as a supersaturated sugar solution with a relatively low protein content, usually ranging between 0.1 and 0.5% [7,8], and a negligible amount of lipids, generally below 0.1% [4]. Honey proteins are primarily enzymes, including glucose oxidase, catalase, and invertase, as well as small peptides and free amino acids derived from nectar and bee secretions [7]. Although minor in proportion, the non-sugar fraction (approximately 3–5%) contains a wide spectrum of bioactive compounds such as phenolic acids, flavonoids, vitamins, minerals, organic acids, and volatile substances that are responsible for the biological and health-promoting properties of honey [5]. The qualitative and quantitative composition of these constituents depends on the botanical and geographical origin of the honey as well as the processing and storage conditions [6,9].

Due to its relatively simple and well-defined composition, honey provides a suitable matrix for studying the effects of enrichment with bioactive ingredients. However, variety selection plays a key role in determining both the physicochemical properties and the technological feasibility of enrichment processes. Among different monofloral varieties, rapeseed honey (*Brassica napus* L.) is most commonly used as a matrix for enrichment due to its low cost, fine-grained crystallization, and relatively stable chemical composition [9,10,11]. Its rapid crystallization allows for efficient creaming, enabling homogeneous dispersion of additives and ensuring high product stability without phase separation during storage [12,13]. These technological characteristics make rapeseed honey an excellent carrier for bioactive compounds in the development of functional honey-based products [8,14,15].

In recent years, there has been a growing interest in spirulina used in food technology for the production of dietary supplements or as feed additives. Spirulina is the commercial name for cyanobacteria of the *Limnospira* (formerly *Arthrospira*) genus, including the *L. platensis* and *L. maxima* species [16], cultivated in warm waters in many countries around the world, whereas Spirulina Pacifica® originates exclusively from Hawaii. This blue-green microalga is recognized for its exceptional nutritional and functional properties [17,18]. Two main commercial forms of spirulina are available on the market: green (powdered sun-dried spirulina) and blue (dry extract of cyanobacteria, containing condensed phycocyanin dye). This pigment is soluble in water but sensitive to high temperature and pH changes, which limits its use for coloring food products [18]. However, the direct addition of microalgal biomass to food formulations is limited due to its large impact on taste, odor, and appearance. It has already been added to, among others, pasta [19], beverages [20], ice creams [21], and supplements [22]. Spirulina has such wide uses due to its rich chemical composition; it contains 50–70% protein in its dry matter, encompassing all essential amino acids, chlorophyll, unsaturated fatty acids (gamma-linolenic acid—GLA) as well as significant amounts of vitamins (especially B group) and minerals such as iron, calcium, magnesium, and potassium [18,23]. It also provides phycobiliproteins, including phycocyanin pigment, responsible for its intense blue color, which exhibits strong antioxidant, anti-inflammatory, and immunomodulatory effects [23,24,25]. Furthermore, due to the absence of cellulose in the cell wall, *L. platensis* demonstrates high digestibility and bioavailability compared with other algal supplements [26,27].

Given these properties, the incorporation of spirulina into rapeseed honey seems to be an innovative approach to developing a functional food with enhanced nutritional, antioxidant, and potential health-promoting attributes. Such enrichment not only aligns with consumer demand for natural, high-value products but also provides a new perspective on the utilization of spirulina as a bioactive food ingredient. Moreover, the high protein and antioxidant content of spirulina makes it particularly attractive for populations with increased nutritional requirements, including athletes, for whom spirulina supplementation has been shown to improve antioxidant capacity, metabolic balance, and physical performance [23,28]. However, combining powdered spirulina with honey and maintaining the homogeneous structure of the product during storage is a real challenge. The only available research on honey enrichment with spirulina was conducted by Güldaş et al. [14], who produced algal honey by feeding bees with sugar syrup with spirulina. The blending of natural honey with spirulina powder used in this study represents a different, more accessible, and novel solution for beekeeping practice.

The innovative aim of this study was to evaluate the effect of spirulina (*L. platensis*) addition on the protein and phenolic content, as well as the physicochemical properties, of the enriched honeys. The stability of the designed products was assessed during storage. For the first time, protein digestibility and the bioavailability of antioxidants were evaluated using an in vitro laboratory digestion model. The proposed process of enriching rapeseed honey is intended to result in a functional product with increased nutritional value and enhanced health-promoting properties, as well as interesting sensory qualities.

## 2. Results and Discussion

**Figure 1:**
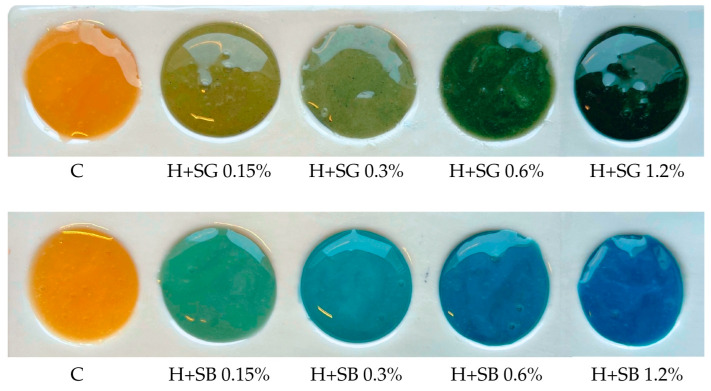
The appearance of the obtained honeys enriched with the addition of green (**top**) and blue (**bottom**) spirulina.

By introducing spirulina additives during rapeseed honey creaming, innovative products were obtained, characterized by an attractive color with growing intensity corelated with the share of the additive introduced to the honey (Figure 1). The texture of the product was creamy and smooth; however, in the case of whole cyanobacteria addition (green product), the small particles of the additive were visible. 

### 2.1. Color of Spirulina-Enriched Honey 

Instrumental color measurements indicated that color parameters in the CIE L*a*b* color space revealed distinct and systematic changes in the visual characteristics of the honeys following the addition of green (SG) and blue (SB) spirulina (Table 1).

The control sample (C) exhibited the highest lightness (L* = 56.06 ± 0.02), typical of rapeseed honey, which naturally shows a light-yellow hue. The incorporation of spirulina resulted in a gradual and statistically significant reduction in the L* parameter, indicating product darkening. For honeys with green spirulina, the lightness decreased from 43.52 (at 0.15% addition) to 13.62 (at 1.2%), whereas in honeys with blue spirulina, the values dropped from 46.57 to 26.53, respectively. These findings demonstrate that both pigments contribute to color darkening, with a stronger lightness reduction observed for the green variant at the highest concentration. The a* parameter, corresponding to the red–green axis, also showed significant changes (*p* < 0.05). Upon the addition of spirulina, the a* values shifted toward negative values, indicating the development of greenish hues, and this effect was much more pronounced for blue spirulina addition. Similarly, the b* parameter, which represents the yellow–blue axis, decreased consistently with increasing additive content. The control sample (b* = 35.23 ± 0.08) showed an intense yellow hue, while both spirulina variants shifted this color toward the blue range, especially for blue spirulina. This trend confirms the dominant coloring influence of phycocyanin; the major blue pigment of cyanobacteria which has high pigmental power even at low concentrations.

The obtained results are consistent with the findings of Güldaş et al. [14], who observed a comparable decrease in lightness and a shift toward green-blue hues in algal honeys obtained by feeding bees with *Spirulina platensis* extract. Similarly, Chaouachi et al. [23] reported that the high phycocyanin and chlorophyll content in *A. platensis* pigments strongly influences color parameters, reducing brightness (L*) and increasing negative a* and b* values in fortified foods. In summary, increasing the concentration of spirulina in honey leads to progressive darkening and a clear chromatic shift from yellow toward green and blue tones. The effect is more pronounced for blue spirulina, confirming its superior pigmenting potential and suitability as a natural colorant for food products, even when used in a matrix with an acidic pH value (4.3 for supplemented rapeseed honey).

### 2.2. Physicochemical Parameters and Antioxidant Activity of Enriched Honeys

An increase in the content of *L. platensis* (green and blue spirulina) in rapeseed honey resulted in significant changes in its physicochemical and bioactive properties after 1 month of storage (Table 2). The pH of the enriched honeys increased gradually with enhancing additive levels (*p* < 0.05), ranging from 4.26 for the control to 4.95 and 5.27 for samples with 1.2% green and blue spirulina, respectively, after one month of storage. This trend was accompanied by a progressive decrease in titratable acidity, by 43% for both green and blue spirulina at the highest concentration compared with the control (*p* < 0.05). This buffering behavior aligns with the observations of Güldaş et al. [14], who reported similar effects in algal honeys as well as with earlier findings describing the ion-exchange and acid–base equilibrium properties of spirulina biomass [23,29]. Electrical conductivity increased proportionally (*p* < 0.05) to the concentration of spirulina, reaching a maximum rise of 53% (green) and 67% (blue) at 1.2% addition. This effect can be attributed to the high mineral content of spirulina, including iron, calcium, and magnesium, which increased ionic activity in the honey matrix [23]. The water content of the enriched honeys slightly decreased with increasing spirulina concentration (by approximately 1%), regardless of the type of spirulina used. The observed decrease can be attributed to the water-binding capacity of algal macromolecules, particularly proteins and polysaccharides, which can interact with water molecules through hydrogen bonds [29]. The reduction in water content is technologically advantageous, as it decreases the risk of fermentation and microbial growth during storage, while also improving the texture and spreadability of the product. Similar trends were reported by Güldaş et al. [14], who observed a decrease in water content and enhanced stability in algal honeys. 

A clear concentration-dependent effect was also observed in antioxidant activity (*p* < 0.05) in the samples stored for one month. For the DPPH assay, the values increased from 3.37 μmol TE/100 g (control) to 8.58 and 10.73 μmol TE/100 g (for 1.2% green and blue spirulina, respectively). A similar, but slightly less pronounced, upward trend was observed for the FRAP and total phenolic content (TPC) values. The reducing capacity, measured by the FRAP assay, showed the highest increase at the maximum spirulina concentration (1.2%), reaching 31% and 105% for green and blue spirulina, respectively. In the case of TPC, the increases were smaller, amounting to 20% and 48% for green and blue spirulina, respectively. These results confirm that the enrichment intensity directly enhances the antioxidant potential of the product, which is consistent with the observations of Dżugan et al. [5] and Štajner et al. [30], who reported similar concentration-dependent relationships in honey fortified with botanical additives.

A comparison of samples enriched with green and blue spirulina demonstrated that the color variant significantly influenced the magnitude of change in all tested parameters. Blue spirulina exhibited a more significant influence on pH, conductivity, and antioxidant activity than the green variant, regardless of concentration or storage time. This difference can be attributed to compositional variability between the two spirulina forms. Blue spirulina is purified phycocyanin, a pigment–protein complex responsible for its characteristic color and strong reducing properties. It acts as a non-enzymatic antioxidant capable of scavenging reactive oxygen species (ROS) and chelating transition metals [23,31]. Consequently, honeys enriched with blue spirulina demonstrated higher total phenolic content and overall antioxidant capacity across all applied assays (DPPH, FRAP, and TPC). In contrast, green spirulina, with a higher chlorophyll and carotenoid fraction, also enhanced the antioxidant profile but to a lesser extent. 

Throughout the six-month storage period, the enriched honeys maintained their physicochemical and bioactive characteristics. No statistically significant changes (*p* > 0.05) were observed in the key parameters of pH, titratable acidity, conductivity, moisture, or antioxidant activity, between the third and sixth month of storage. This indicates high storage stability of both green and blue spirulina-enriched honeys, confirming their resistance to degradation of active compounds. A marginal increase in pH and a decrease in titratable acidity were observed over time, consistent with normal honey maturation processes [32]. Antioxidant capacity remained stable, suggesting that polyphenolic compounds and antioxidants from spirulina exhibit strong resistance to oxidation within the honey matrix. The results are consistent with the findings of Güldaş et al. [14], who reported no significant loss of antioxidant activity in *A. platensis*-enriched algal honey after extended storage. Overall, storage time did not adversely affect the physicochemical integrity or bioactivity of the samples, highlighting the suitability of rapeseed honey as a technologically stable carrier for cyanobacteria-derived bioactive compounds.

**Figure 2:**
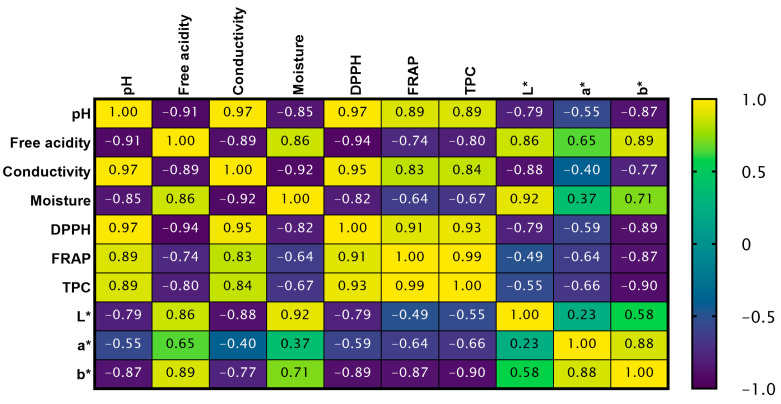
Correlation matrix between all tested parameters.

The correlation results show a clear clustering of parameters, reflecting common trends in changes resulting from spirulina enrichment of honeys (Figure 2). Antioxidant activity (DPPH, FRAP) and polyphenol content (TPC) are strongly positively correlated, confirming that phenolic compounds are associated with antioxidant potential. These parameters are also positively correlated with conductivity, which can result from the occurrence of antioxidant elements in spirulina [18]. Among the color parameters, the b* value exhibits a significant correlation with antioxidant activity and conductivity, indicating that color intensification is associated with an increased content of antioxidant components derived from spirulina.

### 2.3. Protein Content

**Figure 3:**
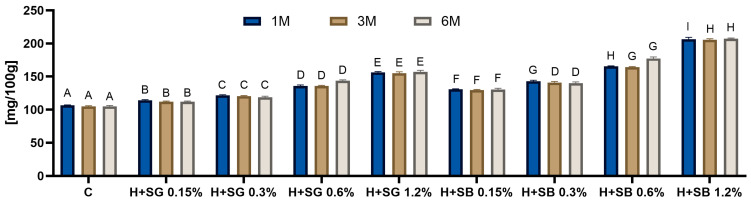
The protein content in spirulina-enriched honeys after 1, 3 and 6 months of storage. ^A,B,C,D,E,F,G,H,I^—means marked with different superscript letters are significantly different (*p* < 0.05) between samples for a given storage time. SG—spirulina green, SB—spirulina blue; 0.15, 0.3, 0.6 and 1.2%—percentage addition of spirulina.

The protein content in the spirulina-enriched samples increased significantly (*p* < 0.05) with increasing spirulina addition, regardless of the type used (green and blue) (Figure 3). Samples containing blue spirulina (SB) consistently showed higher protein concentrations at all addition levels compared to green spirulina (SG) (*p* < 0.05). This difference became particularly evident at the highest supplementation level (1.2%), where honey enriched with blue spirulina reached approximately twice the protein content of the control, while the increase in green spirulina was noticeably smaller (by 95% and 45%, respectively). This phenomenon can be attributed to the biochemical composition of both forms of *L. platensis*. Blue spirulina, as a concentrated phycobiliprotein, strongly increases total protein levels compared to green spirulina, which contains more chlorophyll-related proteins and carotenoids, and contributes less to total soluble protein [33]. 

During the six-month storage period, the protein content of the enriched honeys remained stable. No statistically significant (*p* > 0.05) differences were observed between measurements taken after 1, 3, and 6 months, regardless of the spirulina type or its concentration. The stability of protein content during storage suggests that the honey environment effectively protects spirulina-derived proteins from degradation, confirming the technological suitability of rapeseed honey as a carrier for protein-rich spirulina additives. This finding is consistent with the reports of Bonsignore et al. [34], who highlighted the protective role of low water activity and the antioxidant environment in honey in maintaining the integrity of bioactive compounds during long-term storage. Overall, the enrichment of rapeseed honey with spirulina substantially improved its nutritional value, in terms of increased protein content (up to twice for 1.2% SB addition), confirming that both forms of *L. platensis* serve as effective bioactive enhancers, with blue spirulina demonstrating a more pronounced impact on protein fortification.

### 2.4. Bioaccessibility of Bioactive Components

The concept of bioavailability refers to the fraction of a nutrient or bioactive substance that is potentially absorbed from the gastrointestinal tract and reaches the organs and tissues, and participates in the basic metabolic processes or other biological processes [35]. It is a complex concept that includes four distinct processes: bioaccessibility, absorption, metabolism, and bioactivity. During an in vitro study, bioaccessibility assessment, which involves the digestion of food using selected procedures and the evaluation of the fraction of an ingested biocomponent that becomes accessible for absorption through the epithelial layer of the gastrointestinal tract, is usually performed [35,36]. In the case of native polymers, it is also related to the term digestibility, i.e., the ability of food products to be broken down into low-molecular-weight components under the influence of enzymes. The digestibility of protein can be determined in vitro by assessing the degree of degradation (enzymatic hydrolysis) in laboratory conditions, using a model that is designed to mimic the human or animal digestive system and the individual stages of digestion. The stability and bioaccessibility of the phenolic compounds and antioxidants and the protein digestibility of the control and most-spirulina-enriched honeys (1.2% additive content only) were assessed using simulated in vitro digestion in a stomach and intestine model. The results of the total phenolic content, FRAP parameter, and soluble protein content (Bradford method) are summarized in Table 3.

**Figure 4:**
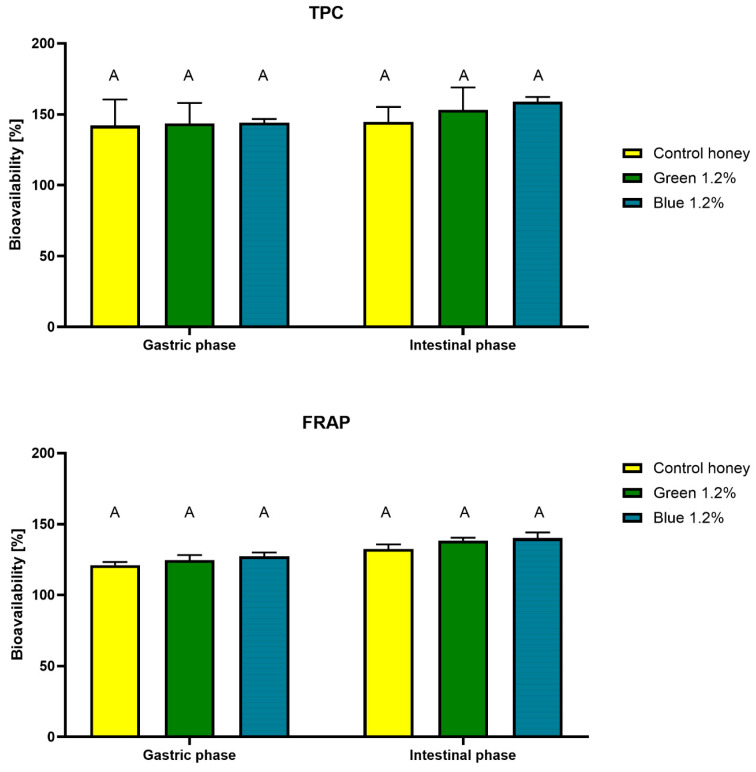
Bioavailability of total phenols (TPC) and antioxidant compounds (FRAP) after in vitro digestion. ^A^—means marked with the same letters do not differ significantly (*p* > 0.05) within digestion stage.

The results obtained using the FRAP and TPC methods indicate that the bioaccessibility of polyphenolic components increases with the progress of digestion, which may be due to a better release of these components from the matrix subjected to enzymatic digestion. The amount of detected polyphenols increases during simulated digestion compared to the undigested fraction (100%), reaching 144.02% in the stomach and 158.89% in the intestine for the blue spirulina (Figure 4). The results for the control honey and the green spirulina did not differ significantly. Similarly, for antioxidants, measured by the FRAP method, bioavailability increased to 124–127% in the stomach and 138–139% in the intestine.

The bioavailability of active ingredients present in honey has been formerly studied, and it has been shown that honey phenolic compounds exhibit high stability during digestion, with some even increasing in concentration. The bioaccessible fraction of phenolic compounds of bracatinga (*Mimosa scabrella*) honeydew honey ranged from 78.2% to 174.38% after digestion [37]. Studies of Alevia et al. [38] have shown that digested honey samples had higher total phenolic content and antioxidant activity compared to pre-digested samples. This indicates that the digestion process can enhance the bioavailability of these compounds. The bioavailability of bioactive components from herb honeys with spirulina was studied by Güldaş et al. [14]. The digestibility values for total phenolic content were 14.37% and 11.18% for algal honey with *L. platensis* and the control honey, respectively. The resulting relationship, i.e., greater in vitro bioavailability of antioxidants from algal honeys enriched with spirulina compared to control honey, is consistent with the results obtained in this study. Similarly, the antioxidant activity (measured by ABTS and CUPRAC methods) after digestion of algal honey was higher compared to unfortified honey [14]. Moreover, Peláez-Acero et al. [39] demonstrated the positive effect of ultrasound pretreatment (10, 20, and 30 min) on the bioaccessibility of the antioxidant fraction of Mexican honeys subjected to in vitro digestion compared to untreated honeys. 

**Figure 5:**
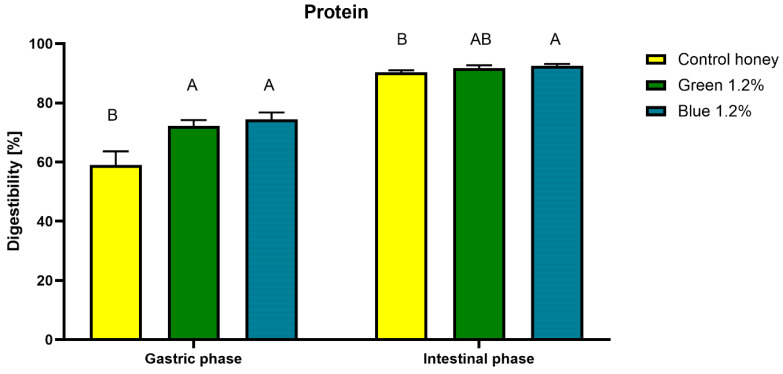
Digestibility of protein in simulated in vitro digestion. ^A,B^—means marked with different letters differ significantly (*p* < 0.05) within digestion stage.

In the case of protein content, a reduced value after in vitro digestion indicates the better digestibility of the protein contained in the tested sample. Protein digestibility results expressed as a percentage of the initial protein content are presented in Figure 5.

**Figure 6:**
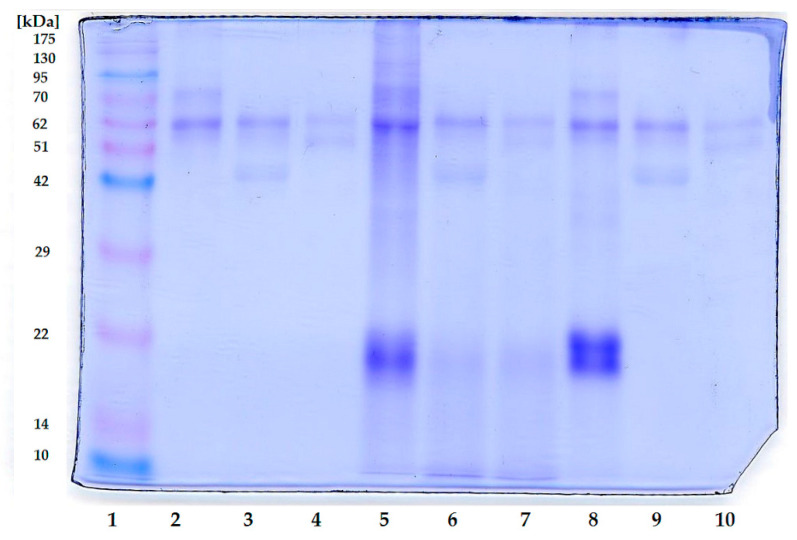
SDS-PAGE gel image. 1—molecular weight marker, 2—rapeseed honey (undigested fraction); 3—rapeseed honey (gastric fraction); 4—rapeseed honey (intestinal fraction); 5—rapeseed honey + green 1.2% (undigested fraction); 6—rapeseed honey + green 1.2% (gastric fraction); 7—rapeseed honey + green 1.2% (intestinal fraction); 8—rapeseed honey + blue 1.2% (undigested fraction); 9—rapeseed honey + blue 1.2% (gastric fraction); 10—rapeseed honey + blue 1.2% (intestinal fraction).

The final protein digestibility results for all three honeys tested were above 90%, with the highest digestibility (92.51%) achieved for the honey with blue spirulina. Protein digestion occurs primarily in the gastric phase under the influence of pepsin, reaching 59.06% at this stage for the control rapeseed honey and a significantly higher percentage for honeys enriched with spirulina, above 72% (Figure 5). The protein profiles of honeys digested in vitro were also examined using the SDS-PAGE method. The results (Figure 6) indicate that in vitro digestion of the analyzed control honey resulted in a gradual reduction in the intensity of the protein bands compared to the undigested honey.

The control rapeseed honey, not subjected to simulated digestion, was characterized by bands above 51 kDa, with the 62 kDa band exhibiting the highest intensity. In the gastric phase, an additional band (about 42 kDa) was observed, most likely derived from pepsin. Subsequent digestion stages resulted in the 70 kDa and 62 kDa bands becoming fainter, indicating the incomplete digestibility of honey-derived proteins. Enriching honey with green or blue spirulina resulted in a greater saturation of the bands present in rapeseed honey and the appearance of new additional bands ranging from about 19 to 22 kDa, specific to *L. platensis*. Furthermore, honey enriched with green spirulina, but not digested, was characterized by higher intensity bands in the 62–175 kDa range. Honey enriched with blue spirulina was also characterized by the presence of additional bands in the 19–22 kDa range. In vitro digestion of the enriched honeys resulted in the disappearance of the lower mass bands and a reduction in the intensity of the bands in the higher molecular mass range, indicating the high digestibility of proteins originating from the spirulina additive already present in the enriched honeys during gastric digestion. Further protein degradation in the simulated intestine resulted in a further disappearance of the bands, indicating further protein digestion in this part of the gastrointestinal tract.

The protein profiles of honeys are generally poor; the main proteins identified by SDS-PAGE are major royal jelly proteins (MRJPs), whose molecular weights are in the range of 45–68 kDa [40,41]. The bands corresponding to proteins with masses of approximately 59 and 70 kDa were previously found to be dominant in the electrophoretic image of rapeseed honeys [33]. The presence of additional protein bands in the SDS-PAGE image originating from cyanobacteria is evidence of enrichment of the initial honey with additional proteins. The additional protein bands observed in spirulina honeys (14–22 kDa) most likely originate from α/β biliprotein subunits [42]. The band at approximately 95 kDa can be assigned to the core membrane linker protein of *L. platensis* [42], although a protein of similar mass has also been noted in honey [43].

Spirulina protein exhibits high in vitro digestibility, with values reported to be over 90% under optimized extraction conditions. This value is higher than for many other plant and algae proteins, including commercial soybean protein isolate [44]. Hence, the use of spirulina as a protein-fortifying agent in honey seems justified. Spirulina contains approximately 60–70% protein, which is higher than most plant-based sources and comparable to animal proteins. Moreover, it is rich in several bioactive compounds, including vitamins and minerals [23]. 

### 2.5. Organoleptic Assessment

**Figure 7:**
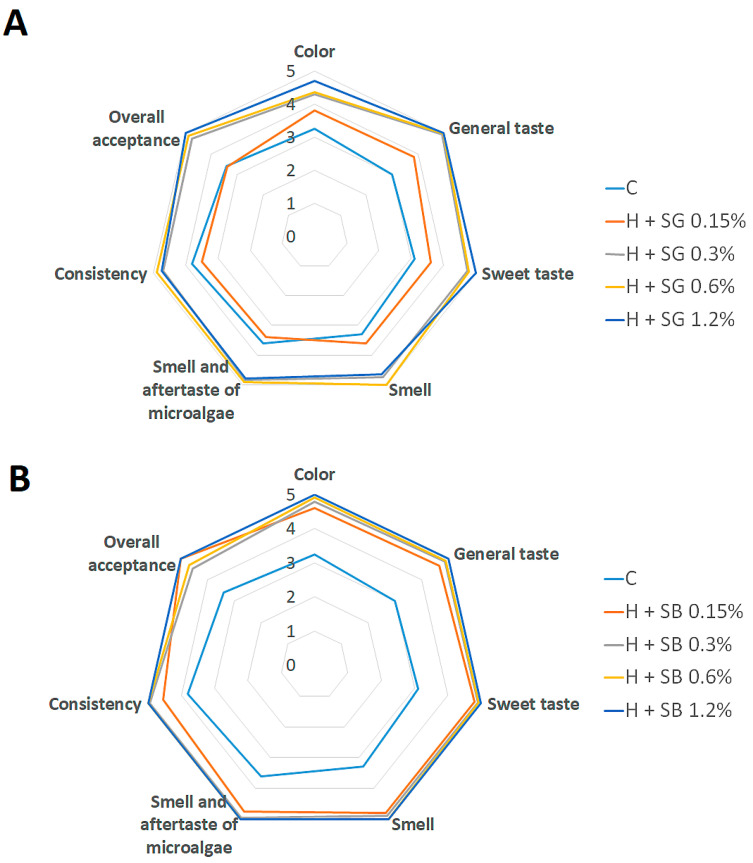
Results of organoleptic assessment of honeys enriched with green (**A**) and blue (**B**) spirulina.

Given that spirulina is a strongly pigmented additive and color plays a key role in consumer perception, the study was expanded to include a complementary sensory evaluation. This allowed verification of whether the chemical and functional improvements observed for spirulina-enriched honeys were accompanied by consumer acceptability. An organoleptic evaluation of the enriched honeys was carried out after 30 days of storage under optimal conditions by ten trained panelists. Given the specific nature of the spirulina-enriched products, additional attributes such as the intensity of the microalgal aroma and aftertaste, as well as the overall sweetness, were included in the set of evaluated parameters. The results are summarized in Figure 7.

Analysis of the panelists’ scores revealed that the blue honey with the highest spirulina concentration (1.2%) achieved the best overall evaluation, receiving the highest rating for color (5.0 points). In contrast, the control sample (without spirulina) obtained the lowest color score (3.25 points). Regarding taste, the most important sensory attribute, the sample enriched with 1.2% blue spirulina again received the highest score and was rated most favorably in terms of aroma (5.0 points from all panelists). When evaluating consistency, the product enriched with 1.2% blue spirulina received the maximum score (5.0 points), indicating a smooth and uniform texture, while the lowest rating (3.5 points) was recorded for honey with 0.15% green spirulina. Because the samples were enriched with spirulina, panelists also evaluated the intensity of the algae-like flavor. The green honey with 0.15% spirulina addition was described as the mildest in this regard, whereas higher concentrations produced a more noticeable but acceptable marine note. To sum up, the sample with 1.2% SB appeared in laboratory analysis as the most improved regarding the tested parameters: it had protein and antioxidant contents of high bioaccessibility and was evaluated as the best by consumers.

The last findings seem to be the most important for implementing the proposed novel sustainable technology in the beekeeping sector, provided that appropriate marketing is launched. The designed product has many advantages that can determine the choice of consumers: good sensory characteristics and evidenced health benefits [45]. Consumers tend to prefer functional foods where the added ingredient is a natural fit with the “carrier” food and generally favor less-processed products. Moreover, consumer knowledge about the functional ingredient spirulina, known as an alternative source of protein, and its specific health benefits is a key predictor of acceptance [27]. Emphasizing this and targeting health-motivated consumer segments are key strategies for introducing a proposed functional product into the diet of various consumer groups, especially children.

## 3. Materials and Methods

Rapeseed honey of a confirmed variety (pollen analysis) was obtained directly from the organic apiary (49°48′22.8″ N 21°32′33.4″ E) in April 2023. Before enrichment, the honey was subjected to a liquefaction process (42 °C, 48 h) using a laboratory dryer (SLN 53 STD, Pol Eco, Wodzisław Śląski, Poland) [8]. To enrich the honey, two dietary supplements: SG-powdered green spirulina (Spirulina Pacifica® Cyanotech Co, distributed by Kenay, Kalisz, Poland) and SB-blue spirulina (100% Phycocyanin powder extract of spirulina originating from Spain. The Organic Lab Nordic, Stockholm, Sweden) purchased online from a certified supplier, were used.

### 3.1. Preparation of Experimental Samples

Rapeseed honey was combined with powdered green spirulina and blue spirulina (a dehydrated powder consisting solely of spirulina extract) in four increasing doses: 0.15%, 0.3%, 0.6% and 1.2% (*w*/*w*), added during the creaming process according to the procedure herein [46]. Briefly, an appropriate mass (150 g after subtracting the amount of added powder) of previously liquefied honey was weighed into 250 mL glass jars, in duplicate for each variant. All samples were inoculated with 1% rapeseed honey crystals, and the appropriate amount of powdered spirulina (blue or green) was added. Each sample was homogenized with a hand mixer (MFQ 3540, Bosh, Munich, Germany) for 4 min at a low speed (60 rpm) at ambient temperature (20 °C), which ensured the distribution of honey crystals and spirulina (experimental samples). The prepared samples were placed in a refrigerator (5 °C) for 24 h to allow crystallization. The creaming process was then carried out for 7 days, during which the samples were mixed twice daily for 4 min each, maintaining constant homogenization parameters. After completion of the creaming stage, the honeys were stored under refrigeration (5 °C) for an additional 7 days to stabilize the structure. Then the samples were stored at room temperature (20 ± 2 °C), away from sunlight and moisture. Analyses were performed after 1, 3 and 6 months of storage.

### 3.2. Physicochemical Parameters 

The physicochemical parameters of the enriched honeys were determined according to standard analytical procedures for honey as described in detail earlier [46]. The pH was measured using 20% (*w*/*w*) honey solutions using a SevenCompact™ S210 pH meter (Mettler Toledo, Columbus, OH, USA), and free acidity was determined by titration with 0.1 M NaOH solution to an endpoint of pH 8.3. The electrical conductivity of the 20% (*w*/*w*) honey solutions was measured using a CP-401 conductometer (Elmetron, Zabrze, Poland). Water content was determined refractometrically using a HI96800 digital refractometer (Hanna Instruments, Woonsocket, RI, USA).

### 3.3. Color Analysis

Color measurements (L*, a*, b*) were made using a CR 5 colorimeter (Konica Minolta, Tokyo, Japan) in reflection mode with SCE (Specular Component Excluded), at an angle of 10°/D 65, with an aperture diameter of 30 mm according to manufacturer instructions experimentally adapted to honey. Before the measurements, the device was calibrated for white and black according to the manufacturer’s instructions. A 5 mm thick layer of each honey was applied to a 55 mm diameter polystyrene dish. Three replicates were performed for each sample.

### 3.4. Total Phenolic Content and Antioxidant Capacity

Total phenolic content was measured using the Folin–Ciocalteu method, while antioxidant capacity was measured using the DPPH (2,2-diphenyl-1-picrylhydrazyl) and FRAP (ferric reducing–antioxidant power) methods for 20% honey solutions. Detailed procedures are described in [47].

### 3.5. Soluble Protein

The protein content of honey samples was determined using the Bradford method, following the procedure described by Dżugan et al. [48]. To 20 μL of each honey solution, 1000 μL of Bradford reagent (G-250) was added, and the mixtures were incubated at room temperature for 5 min. Absorbance was then measured at 595 nm using a Biomate 3 spectrophotometer (Thermo Scientific, Waltham, MA, USA). Protein concentration was calculated based on the calibration curve prepared for bovine serum albumin in the range of 0–100 μg/sample (y = 0.0555x, r^2^ = 0.998).

### 3.6. In Vitro Digestion of the Fortified Honeys

The protein digestibility and bioaccessibility of polyphenolic compounds were assessed using a two-stage simulated digestion model, including the stomach and intestine stages using the method modified by Dżugan et al. [49]. For the in vitro digestion experiment, three honey samples were selected: control (C) and samples enriched with green (G) and blue (B) spirulina at the highest 1.2% concentration. As the undigested control, 2 mL of 20% (*w*/*v*) aqueous honey solution was used. 

Gastric phase (stage I): 2.5 mL of pepsin was added to each honey sample, and the pH was adjusted to 2 with 5 M HCl. The mixtures were incubated at 37 °C for 2 h, shaking every 15 min. After neutralization to pH 7.2 with 1 M NaHCO_3_, 2 mL of gastric fractions were collected.

1. Intestinal phase (stage II): to the gastric digests obtained in stage I, 12.5 mL of a bile and pancreatin mixture was added. Samples were incubated at 37 °C for 2 h with intermittent shaking. Subsequently, 2 mL of intestinal fractions were collected.
2. The undigested (U), gastric (G), and intestinal (I) fractions were analyzed for polyphenolic compounds, and antioxidant capacity, as well as protein content, using the methods described previously in Section 3.4 and Section 3.5. The bioaccessibility index (BI, %) was calculated according to the following Formula (1):
  (1) $$\mathrm{BI}\;\left[\%\right]=\frac{C_{\mathrm{digested}}}{C_{\mathrm{undigested}}}\times 100\%$$
  where
  BI [%]—bioaccessibility index of bioactive compounds;
  C_digested_—concentration of the bioactive compound in the digested phase (gastric/intestinal);
  C_undigested_—concentration of the bioactive compound in the undigested phase.
  Soluble protein digestibility was calculated using the same formula as 100%−BI.

### 3.7. Protein Profiling by SDS-PAGE

The protein profile of the samples subjected to simulated digestion was analyzed by denaturing electrophoresis (SDS-PAGE) according to the procedure described by Dżugan et al. [43], with some modifications. Immediately before loading the samples onto the gel, 30 µL of each sample (fraction after in vitro digestion) was heated for 5 min at 100 °C with 15 µL of standard Laemmli buffer. After cooling, the denatured samples (20/21.5/24 µL, depending on the fraction) were loaded onto a 12.5% gel prepared 24 h earlier. Electrophoresis was performed, initially at 50 V (15 min), and then at 150 V for 2 h using a Bio-Rad PowerPac 3000 (Bio-Rad Laboratories, Hercules, CA, USA). Prestained ROTI®Mark BI-PINK (Carl Roth GmbH, Karlsruhe, Germany) was used as a molecular weight marker as a standard. After electrophoresis, the gel was stained with colloidal Coomassie Brilliant Blue G-250 for 12 h, and then the gel was destained for 24 h with a destaining solution consisting of ethanol, acetic acid, and water (4:1:15) to remove the stain. The gel was scanned using an Epson Perfection V850 Pro scanner (Simatupang, Jakarta Selatan, Indonesia).

### 3.8. Organoleptic Evaluation

Consumer acceptance of the honey samples enriched with spirulina powder was performed using the 5-point hedonic scale: from 1 (dislike extremely) to 5 (like extremely), as described by Habryka et al. [50]. Honey samples (50 g) in glass jars were given to a 10-person sensory panel, with approximately 2 g taken for taste testing, and mineral water was used as a neutralizer between samples. The organoleptic evaluation included color, taste, consistency, and smell, as well as the overall note. 

### 3.9. Statistical Analysis

All analyses were performed in triplicate unless otherwise indicated. Results are presented as mean ± standard deviation (SD). Statistically significant differences between different concentrations of spirulina, different periods of storage, and the kind of spirulina (blue vs. green) were calculated using multivariate analysis of variance (ANOVA) (*p* < 0.05), followed by Tukey’s test [46]. The relationships between the analyzed parameters were evaluated using Pearson’s correlation coefficients. The statistical analyses were performed, and the graphs were generated using GraphPad Prism 10 software (GraphPad Software, Boston, MA, USA).

## 4. Conclusions

The conducted study demonstrated that the enrichment of rapeseed honey with spirulina (*Limnospira platensis*) significantly influenced the sensory and nutritional properties of the obtained novel products. Spirulina-enriched rapeseed honeys were characterized, depending on the amount of the additive, by increased protein content, higher antioxidant activity, and good storage stability. In all of the tested samples, blue spirulina (an extract of cyanobacteria) proved to be a more effective ingredient than the green variant (whole cyanobacteria). Moreover, the increase in protein content was positively correlated with improved protein digestibility, as confirmed by in vitro digestion supported with SDS-PAGE analysis. Overall, the results indicate that spirulina, particularly the blue form, can serve as an effective natural additive for enhancing the health-promoting value of honey and buffering its acidity. The spirulina-enriched honeys showed an interesting sensory characteristic that can positively influence the consumer’s choice. It can therefore be recommended as a new, functional product with proven health-promoting properties, intended for dietary supplementation. Due to the experiment being conducted on a small laboratory scale, it is necessary to extend the research scale and to confirm the bioavailability of nutrients and bioactive spirulina-delivered components in an *in vivo* model.

## Figures and Tables

**Table 1:** CIE L*a*b* color measurements of control (C) and spirulina-enriched honeys (SG—green spirulina; SB—blue spirulina) after 6 months of storage.

	L*	a*	b*
C	56.06 ± 0.02 ^A^	8.14 ± 0.01 ^A^	35.23 ± 0.08 ^A^
H + SG 0.15%	43.52 ± 0.02 ^C^	−1.98 ± 0.01 ^B^	20.68 ± 0.03 ^B^
H + SG 0.3%	40.35 ± 0.06 ^E^	−4.63 ± 0.02 ^D^	14.54 ± 0.04 ^C^
H + SG 0.6%	21.71 ± 0.04 ^H^	−4.87 ± 0.04 ^E^	9.79 ± 0.13 ^D^
H + SG 1.2%	13.62 ± 0.05 ^I^	−2.85 ± 0.02 ^C^	5.02 ± 0.07 ^F^
H + SB 0.15%	46.57 ± 0.09 ^B^	−15.02 ± 0.04 ^F^	7.39 ± 0.03 ^E^
H + SB 0.3%	40.59 ± 0.01 ^D^	−18.92 ± 0.02 ^I^	−3.23 ± 0.02 ^G^
H + SB 0.6%	30.59 ± 0.06 ^F^	−17.76 ± 0.04 ^H^	−9.27 ± 0.01 ^H^
H + SB 1.2%	26.53 ± 0.06 ^G^	−15.33 ± 0.04 ^G^	−15.18 ± 0.02 ^I^

^A,B,C,D,E,F,G,H,I^—means within the column marked with different superscript letters are significantly different (*p* < 0.05).

**Table 2:** Physicochemical parameters and antioxidant activity of spirulina-enriched honeys after 1, 3 and 6 months of storage.

Honey Analysis after 1 Month of Storage									
Parameter	Control	+green spirulina				+blue Spirulina			
		The content of spirulina additive							
		0.15%	0.3%	0.6%	1.2%	0.15%	0.3%	0.6%	1.2%
Physicochemical parameters									
pH	4.26 ± 0.01 ^A^	4.35 ± 0.1 ^B^	4.44 ± 0.01 ^C^	4.58 ± 0.01 ^D^	4.95 ± 0.02 ^E^	4.44 ± 0.01 ^C^	4.60 ± 0.01 ^D^	4.94 ± 0.02 ^E^	5.27 ± 0.03 ^F^
Free acidity [mval/kg]	15.05 ± 0.07 ^A^	14.35 ± 0.21 ^B^	12.15 ± 0.21 ^C^	10.20 ± 0.14 ^D^	8.60 ± 0.14 ^E^	12.85 ± 0.07 ^F^	10.3 ± 0.14 ^D^	9.75 ± 0.07 ^G^	8.65 ± 0.07 ^E^
Conductivity [mS/cm]	157.10 ± 0.02 ^A^	172.83 ± 0.25 ^B^	187.94 ± 0.21 ^C^	203.11 ± 0.08 ^D^	240.09 ± 0.02 ^E^	175.03 ± 0.07 ^F^	190.15 ± 0.15 ^G^	215.02 ± 0.02 ^H^	262.20 ± 0.18 ^I^
Moisture [%]	17.3 ± 0.1 ^A^	17.1 ± 0.2 ^AB^	16.9 ± 0.1 ^BC^	16.7 ± 0.1 ^CD^	16.4 ± 0.1 ^AB^	17.1 ± 0.2 ^BC^	16.9 ± 0.1 ^CD^	16.7 ± 0.1 ^D^	16.4 ± 0.2 ^D^
Antioxidant activity									
DPPH [μmol TE/100 g]	3.37 ± 0.24 ^A^	3.93 ± 0.73 ^A^	4.82 ± 0.35 ^B^	6.17 ± 0.43 ^C^	8.58 ± 0.24 ^E^	5.62 ± 0.26 ^C^	6.19 ± 0.47 ^C^	9.06 ± 0.30 ^D^	10.73 ± 0.60 ^D^
FRAP [μmol TE/100 g]	19.21 ± 0.23 ^A^	20.70 ± 0.27 ^B^	21.45 ± 0.38 ^C^	22.31 ± 0.62 ^D^	25.20 ± 0.13 ^E^	24.71 ± 0.14 ^E^	26.35 ± 0.07 ^F^	31.09 ± 0.15 ^G^	39.47 ± 0.07 ^H^
TPC [mg GAE/100 g]	7.99 ± 0.26 ^A^	8.30 ± 0.19 ^B^	8.50 ± 0.19 ^BC^	8.69 ± 0.14 ^C^	9.55 ± 0.12 ^D^	9.78 ± 0.13 ^DE^	10.04 ± 0.07 ^E^	10.51 ± 0.08 ^F^	11.79 ± 0.17 ^G^
Honey analysis after 3 months of storage									
Parameter	Control	+green spirulina				+blue spirulina			
		The content of spirulina additive							
		0.15%	0.3%	0.6%	1.2%	0.15%	0.3%	0.6%	1.2%
Physicochemical parameters									
pH	4.31 ± 0.02 ^A^	4.36 ± 0.01 ^B^	4.48 ± 0.01 ^C^	4.59 ± 0.02 ^D^	4.95 ± 0.01 ^E^	4.45 ± 0.01 ^F^	4.62 ± 0.01 ^G^	4.97 ± 0.01 ^E^	5.29 ± 0.01 ^H^
Free acidity [mval/kg]	15.10 ± 0.14 ^A^	13.3 ± 0.14 ^B^	11.90 ± 0.14 ^C^	10.15 ± 0.07 ^D^	8.55 ± 0.07 ^E^	12.85 ± 0.07 ^F^	10.25 ± 0.07 ^D^	9.65 ± 0.07 ^G^	8.60 ± 0.14 ^E^
Conductivity [mS/cm]	156.67 ± 0.59 ^A^	171.93 ± 0.06 ^B^	187.87 ± 0.06 ^C^	222.96 ± 0.03 ^D^	240.01 ± 0.03 ^E^	174.98 ± 0.03 ^F^	190.01 ± 0.03 ^G^	224.99 ± 0.02 ^H^	262.10 ± 0.06 ^I^
Moisture [%]	17.2 ± 0.1 ^A^	17.0 ± 0.1 ^AB^	16.9 ± 0.1 ^BC^	16.5 ± 0.1 ^DE^	16.4 ± 0.1 ^AB^	17.0 ± 0.1 ^BC^	16.8 ± 0.1 ^CD^	16.6 ± 0.1 ^DE^	16.3 ± 0.2 ^E^
Antioxidant activity									
DPPH [μmol TE/100 g]	4.00 ± 0.10 ^A^	4.77 ± 0.77 ^AB^	5.60 ± 0.28 ^BC^	7.83 ± 0.71 ^D^	9.34 ± 0.56 ^E^	6.38 ± 0.11 ^CF^	6.87 ± 0.58 ^F^	9.70 ± 0.14 ^E^	11.34 ± 0.66 ^E^
FRAP [μmol TE/100 g]	19.69 ± 0.95 ^A^	21.69 ± 0.38 ^B^	21.98 ± 0.67 ^BC^	22.91 ± 0.78 ^C^	26.20 ± 0.45 ^DE^	25.32 ± 0.31 ^E^	27.09 ± 0.55 ^D^	32.04 ± 0.58 ^F^	40.75 ± 0.40 ^G^
TPC [mg GAE/100 g]	8.29 ± 0.10 ^A^	8.47 ± 0.24 ^B^	8.66 ± 0.09 ^B^	8.99 ± 0.15 ^B^	9.90 ± 0.18 ^C^	9.93 ± 0.23 ^C^	10.22 ± 0.12 ^C^	11.06 ± 0.15 ^D^	12.28 ± 0.18 ^E^
Honey analysis after 6 months of storage									
Parameter	Control	+green spirulina				+blue spirulina			
		The content of spirulina additive							
		0.15%	0.3%	0.6%	1.2%	0.15%	0.3%	0.6%	1.2%
Physicochemical parameters									
pH	4.33 ± 0.02 ^A^	4.44 ± 0.01 ^B^	4.56 ± 0.01 ^C^	4.92 ± 0.01 ^D^	4.96 ± 0.03 ^D^	4.53 ± 0.02 ^C^	4.70 ± 0.02 ^E^	5.15 ± 0.01 ^F^	5.32 ± 0.03 ^G^
Free acidity [mval/kg]	14.54 ± 0.02 ^A^	13.37 ± 0.04 ^B^	11.69 ± 0.04 ^C^	9.77 ± 0.01 ^D^	8.40 ± 0.04 ^E^	11.25 ± 0.03 ^F^	10.27 ± 0.05 ^G^	8.64 ± 0.01 ^H^	8.57 ± 0.03 ^EH^
Conductivity [mS/cm]	156.67 ± 0.15 ^A^	172.57 ± 0.21 ^B^	187.77 ± 0.15 ^C^	231.70 ± 0.26 ^D^	245.53 ± 0.43 ^E^	173.97 ± 0.65 ^B^	190.73 ± 0.25 ^C^	234.33 ± 1.29 ^D^	276.13 ± 0.41 ^E^
Moisture [%]	17.15 ± 0.07 ^A^	17.00 ± 0.14 ^AB^	16.85 ± 0.07 ^BC^	16.45 ± 0.07 ^DE^	16.35 ± 0.07 ^DE^	16.95 ± 0.07 ^AB^	16.75 ± 0.07 ^BC^	16.60 ± 0.14 ^CD^	16.25 ± 0.21 ^E^
Antioxidant activity									
DPPH [μmol TE/100 g]	3.95 ± 0.37 ^A^	4.60 ± 0.27 ^AB^	5.67 ± 0.17 ^B^	8.19 ± 1.50 ^D^	10.56 ± 0.50 ^E^	6.86 ± 0.42 ^C^	7.57 ± 0.66 ^CD^	10.64 ± 0.36 ^E^	12.74 ± 0.50 ^F^
FRAP [μmol TE/100 g]	20.34 ± 0.58 ^A^	21.47 ± 0.37 ^B^	22.31 ± 0.13 ^C^	24.59 ± 0.77 ^D^	28.25 ± 0.65 ^E^	25.73 ± 0.32 ^D^	27.18 ± 0.31 ^E^	35.02 ± 0.42 ^F^	44.71 ± 0.41 ^G^
TPC [mg GAE/100 g]	8.24 ± 0.19 ^A^	8.74 ± 0.08 ^B^	9.05 ± 0.08 ^B^	9.44 ± 0.43 ^C^	11.14 ± 0.20 ^D^	10.19 ± 0.11 ^E^	10.49 ± 0.07 ^E^	12.00 ± 0.06 ^F^	14.50 ± 0.20 ^G^

^A,B,C,D,E,F,G,H,I^—means within the row marked with different superscript letters are significantly different from each other (*p* < 0.05).

**Table 3:** Total phenolic content, FRAP antioxidant potential and protein content in honeys enriched at individual stages of in vitro digestion compared to control samples.

**Stage**	**Control H**	**H + 1.2% SG**	**H + 1.2% SB**
	TPC [mg GAE/100 g]		
Before digestion	20.24 ± 2.54 ^b,B^	25.47 ± 2.77 ^ab,B^	28.52 ± 0.45 ^a,C^
Gastric phase	28.43 ± 0.39 ^c,A^	36.30 ± 0.19 ^b,A^	41.08 ± 0.70 ^a,B^
Intestinal phase	29.09 ± 1.41 ^c,A^	38.69 ± 0.49 ^b,A^	45.32 ± 0.23 ^a,A^
FRAP [µmol TE/100 g]			
Before digestion	38.27 ± 0.34 ^c,C^	47.59 ± 1.17 ^b,C^	59.32 ± 0.68 ^a,C^
Gastric phase	46.33 ± 0.52 ^c,B^	59.28 ± 0.24 ^b,B^	75.45 ± 0.71 ^a,B^
Intestinal phase	50.64 ± 1.05 ^c,A^	65.79 ± 0.79 ^b,A^	83.03 ± 1.65 ^a,A^
Protein [mg/100 g]			
Before digestion	199.88 ± 8.90 ^c,A^	299.92 ± 2.79 ^b,A^	453.81 ± 16.53 ^a,A^
Gastric phase	81.56 ± 5.53 ^b,B^	83.28 ± 5.85 ^b,B^	115.96 ± 7.16 ^a,B^
Intestinal phase	19.40 ± 0.92 ^b,C^	26.44 ± 2.84 ^b,C^	33.95 ± 2.70 ^a,C^

^a,b,c^—means within a row marked with different lowercase letters differ significantly (*p* < 0.05) for a given digestion stage. ^A,B,C^—means within a column marked with different uppercase letters differ significantly (*p* < 0.05) for a given parameter.

## Data Availability

The original contributions presented in this study are included in the article. Further inquiries can be directed to the corresponding author.

## Abbreviations

The following abbreviations are used in this manuscript:

EFSA	European Food Safety Authority
FDA	Food and Drug Administration
WHO	World Health Organization
GLA	Gamma-Linolenic Acid
SG	Spirulina Green
SB	Spirulina Blue
SCE	Specular Component Excluded
DPPH	2,2-Diphenyl-1-Picrylhydrazyl
FRAP	Ferric Reducing–Antioxidant Power
U	Undigested
G	Gastric
I	Intestinal
BI	Bioaccessibility Index
TE	Trolox Equivalent
TPC	Total Phenolic Content
ROS	Reactive Oxygen Species
ABTS	2,2′-Azinobis(3-Ethylbenzothiazoline-6-Sulfonic Acid)
CUPRAC	Cupric Reducing Antioxidant Capacity
MRJPs	Major Royal Jelly Proteins
